# Promoting Active Communities in a Culture of Distracted Driving

**Published:** 2011-12-15

**Authors:** Matthew Lee Smith, Mark E. Benden, Chanam Lee

**Affiliations:** Department of Health Promotion and Behavior, College of Public Health, University of Georgia. Dr. Smith is also affiliated with the Texas A&M Health Science Center School of Rural Public Health in College Station, Texas; Department of Environmental and Occupational Health, Texas A&M Health Science Center School of Rural Public Health, College Station, Texas; Department of Landscape Architecture and Urban Planning, Texas A&M University, College Station, Texas

Efforts to improve health outcomes through behavioral modification are often complicated by external factors that may thwart success and introduce potential harm. Factors associated with traffic safety present a contemporary challenge to efforts to promote physical activity. One example is the difficulty of encouraging pedestrian-based physical activity because of the growing prevalence of distracted driving.

## Promoting Active Communities

Increasing physical activity is associated with reduced risk for chronic diseases, including obesity, diabetes, cardiovascular disease, certain cancers, and depression (1). In addition to personal and social factors, many elements and features of the built environment (ie, the physical infrastructure related to transportation in a given community) have been linked with physical activity behaviors, particularly walking and bicycling (2). A growing number of municipalities have adopted policies to improve their built environments to support physical activity and active living (eg, more sidewalks, bicycle lanes on roadways) (3).

Many policies and interventions target streets as multifunctional settings for active transportation and physical activity because they are modifiable public infrastructures that most residents use daily. Examples include the Complete Streets initiative (4) and Safe Routes to School (SRTS) program (5). SRTS was allocated federal funds to improve street-related infrastructure involving sidewalks, bicycle lanes, and crosswalks around schools (6). The Complete Streets initiative has been adopted by various local and state governments, and 70 such policies were enacted in 2010. Complete Streets is geared toward modifying communities to become more pedestrian-friendly through integrated transportation systems that support all modes of travel (4). The Complete Streets and SRTS initiatives seek to improve safety among all road users and to promote walking and bicycling. *Healthy People 2020* promotes physical activity as a primary objective and specifies active transportation (walking and bicycling), and community- and street-scale environmental policies to enhance access to physical activity opportunities (7). Development and modification of streets and adjacent structures is convenient and shows promise in furthering *Healthy People 2020* objectives.

## Roadway Safety and Distracted Driving

Although streets have become safer for drivers, pedestrians and bicyclists are especially vulnerable to acute injury and death on roadways. Despite overall decreases in roadway-related deaths, the reduction rate for pedestrian deaths (14%) is only half the rate for vehicular deaths (27%) (8). Evidence suggests that street design features such as traffic-calming devices, raised medians, sidewalks, and crosswalks can enhance safety for pedestrians and bicyclists (9); however, only a small proportion of US streets incorporate such safety features, and roadway design accounts for only a portion of overall roadway safety. Road users' behaviors, especially those of drivers, are key determinants of roadway safety.

In 2009, the National Highway Traffic Safety Association reported that 5,474 people were killed and 448,000 people were injured as a result of distracted driving (10). Distracted driving is engaging in activities while operating a motor vehicle that detract from the attention given to driving. Examples of distracted driving include, but are not limited to, changing the radio station, programming global positioning system devices, consuming food or beverages, grooming, reading, and using a cellular telephone. Research indicates that distracted driving is a growing trend (11). Among distracted driving–related crashes attributed to cellular telephone use, 18% resulted in death while only 4% resulted in injury in 2009. Furthermore, portable technology device use among pedestrians and bicyclists has been associated with automobile-related fatalities in situations in which the pedestrian or bicyclist was determined to be at fault (10).

## Cellular Telephone Saturation: Driving With Technology

The availability and affordability of mobile communication technology is increasing. The Highway Loss Data Institute (HLDI) Bulletin estimated that approximately 90% of the American population owns a cellular telephone, and approximately 30% of these people are smartphone users (12). Furthermore, the HLDI Bulletin estimates that the number of monthly text messages sent has risen from approximately 14 million in 2000 to more than 150 billion in 2010, and could increase 50-fold by the year 2020 (12). Today's cellular telephones enable drivers and pedestrians alike to instantly access the Internet for various activities including online shopping, social networking, and e-mailing. These same devices allow us to simultaneously listen to music, have a conversation with a family member, and read e-mail while driving, walking, or bicycling between destinations. Although at least 30 states have now made it illegal to use hand-held devices for any purpose while driving, those laws have not resulted in reduced collision claims or crash reductions (12). So, what happens when distracted pedestrians and bicyclists meet motorists driving in the same condition?

Health professionals continue to promote physical activity and the development of active communities, which include environmental modifications such as SRTS that encourage residents to become pedestrians and bicyclists. Such efforts have been successful, are gaining popularity, and are receiving support by governmental agencies (via funding streams), community-based entities, and constituents. In the future, we can also anticipate growing pedestrian traffic in previously unused and underutilized areas. Simultaneously, rising trends of distracted driving must be acknowledged. These independently rising growth curves are on a trajectory to intersect, with implications for markedly increased injuries and deaths associated with automobile-pedestrian interactions.

## Questions Without Definitive Answers

The ongoing public health movement encouraging people to walk and bicycle is complicated by technological movements to provide Americans with more technological distractions. When attempting to protect the public and deter automobile-pedestrian interactions, the following questions arise about where to focus efforts (ie, which discipline or health sector) and about which evidence-based strategies to use: Should the public continue to be encouraged to walk/bicycle in unsafe environments? Should funding and efforts to improve roadway design be increased? Should environmental modifications be made in areas with heavy traffic and crash histories? Should laws restricting cellular telephone use including "cell-free zones" for drivers and pedestrians be enforced more rigidly? How should behavioral limits on individual smartphone use while driving motor vehicles be fostered?

## Success Via Legislative Controls

According to the US Department of Transportation, 34 states have enacted legal bans against text messaging while driving, 9 of which have placed overall bans on hand-held cellular telephone use (13). Additionally, states and local municipalities have sanctioned legislation to enable pedestrians, bicyclists, and motorists to more safely share roadways. For example, many states have enacted laws regulating the distance in which motorists pass or follow bicyclists. Although these laws primarily regulate the actions of motorists, legislative actions also exist to ensure that bicyclists use hand signals, protective equipment (eg, helmets), and reflectors or lights at night.

## A Multidisciplinary Approach

As roadways become increasingly unsafe for pedestrians, bicyclists, and motorists due to distracted driving, active communities and physical activity promotion continue to bring about the intended health benefits needed for healthy living. Potential dangers and risks involved in physical activity on roadways should be discussed by experts in various agencies and community sectors. Multidisciplinary collaboration should be emphasized among public health, urban development and planning, traffic safety, and representatives of other related fields, including the communications technology sector and commercial automotive industry. Immediate efforts are needed to educate both the pedestrian and the driver, with the intent to modify unsafe behaviors and to continue improving unsafe street environments. Secondary to these efforts, proper technological solutions should be pursued and supported with expertise, data, and translational research. Empirical studies and funding supports are needed to explore and identify innovative approaches to curb distracted driving, enabling pedestrians to engage in physical activity to reduce risks for obesity and associated chronic conditions while also safely reaching their destinations.
